# Attentional Bias, Pupillometry, and Spontaneous Blink Rate: Eye Characteristic Assessment Within a Translatable Nicotine Cue Virtual Reality Paradigm

**DOI:** 10.2196/54220

**Published:** 2024-06-27

**Authors:** Kelly Elizabeth Courtney, Weichen Liu, Gianna Andrade, Jurgen Schulze, Neal Doran

**Affiliations:** 1 Department of Psychiatry, University of California, San Diego, La Jolla, CA, United States; 2 Department of Computer Science and Engineering, University of California, San Diego, La Jolla, CA, United States; 3 Veterans Affairs San Diego Healthcare System, La Jolla, CA, United States

**Keywords:** nicotine, craving, cue exposure, virtual reality, attentional bias, pupillometry, spontaneous blink rate, eye-tracking, tobacco, VR, development, addiction, eye, pupil, craving, biomarker, biomarkers, tobacco product

## Abstract

**Background:**

Incentive salience processes are important for the development and maintenance of addiction. Eye characteristics such as gaze fixation time, pupil diameter, and spontaneous eyeblink rate (EBR) are theorized to reflect incentive salience and may serve as useful biomarkers. However, conventional cue exposure paradigms have limitations that may impede accurate assessment of these markers.

**Objective:**

This study sought to evaluate the validity of these eye-tracking metrics as indicators of incentive salience within a virtual reality (VR) environment replicating real-world situations of nicotine and tobacco product (NTP) use.

**Methods:**

NTP users from the community were recruited and grouped by NTP use patterns: nondaily (n=33) and daily (n=75) use. Participants underwent the NTP cue VR paradigm and completed measures of nicotine craving, NTP use history, and VR-related assessments. Eye-gaze fixation time (attentional bias) and pupillometry in response to NTP versus control cues and EBR during the active and neutral VR scenes were recorded and analyzed using ANOVA and analysis of covariance models.

**Results:**

Greater subjective craving, as measured by the Tobacco Craving Questionnaire–Short Form, following active versus neutral scenes was observed (*F*_1,106_=47.95; *P*<.001). Greater mean eye-gaze fixation time (*F*_1,106_=48.34; *P*<.001) and pupil diameter (*F*_1,102_=5.99; *P*=.02) in response to NTP versus control cues were also detected. Evidence of NTP use group effects was observed in fixation time and pupillometry analyses, as well as correlations between these metrics, NTP use history, and nicotine craving. No significant associations were observed with EBR.

**Conclusions:**

This study provides additional evidence for attentional bias, as measured via eye-gaze fixation time, and pupillometry as useful biomarkers of incentive salience, and partially supports theories suggesting that incentive salience diminishes as nicotine dependence severity increases.

## Introduction

Automatic appetitive motivational processes are emphasized as critical components in the development and maintenance of substance addiction (eg, dual-process theories [1,2], incentive salience theory [3,4], Tiffany’s model [5], and incentive-habit model [6]). Preclinical and human investigations frequently rely on the use of cue exposure paradigms to elicit these motivational processes in the laboratory. The cue exposure paradigm is largely grounded in associative learning principles, which posit that repeated pairing of specific stimuli and substance consumption produces conditioned reinforcement such that the stimuli become conditioned cues capable of eliciting motivational or incentive salience for the substance [7]. Incentive salience can be a conscious or unconscious process and is defined as the motivation for a reward resulting from the integration of one’s current physiological state and previously learned associations about the reward cue [8]. Subjective craving for substances is thought to reflect the conscious product of high levels of incentive salience [3,4].

Despite this conceptual coherence, a lack of ecological validity in traditional cue exposure paradigms limits our ability to accurately test and interpret incentive salience outcomes. Attempts have been made to improve the potency of cues and the ecological validity of cue-reactivity designs (eg, [9,10]), yet cue exposure studies typically present the cues in isolation, outside of the context of usual use in natural environments (eg, 2D images or single cigarettes). This isolation of cues limits the ability to invoke a *true* craving state in the lab [11,12] and potentially contributes to poor generalization to the real world [13]. Through greater immersion and interaction within typical contexts of use (eg, the presence of others within a setting where the substance is commonly taken), paradigms using virtual reality (VR) technology have greatly enhanced our ability to elicit craving for various substances in the laboratory [12,14-18], including tobacco [19-22]. Further, VR cue exposure paradigms show great promise as treatment platforms by promoting individualized and accessible care, and allowing for the experience of social immersion and reaction to cues within relevant contexts [23]. Thus, VR cue exposure paradigms represent generalizable tasks with substantial potential for utilization within addiction-related research and clinical settings.

Recent technological advances in VR implementation also allow for precise inline assessment of eye-related measures during cue exposure. The integration of eye-tracking technology into the VR headset is a substantial improvement from previous eye-tracking applications that require inadequate camera placement for precision eye tracking, resulting in partial blockage of the field of view. With this improved technology, it is possible to extract several eye-related measurements that are theoretically related to automatic appetitive motivational processes such as incentive salience and subjective craving; these are attentional bias, pupillary responses, and spontaneous eyeblink rate (EBR).

Attentional bias, or the allocation of a disproportionate amount of time attending to substance-related stimuli relative to neutral stimuli, is thought to either cause or index critical processes responsible for substance-seeking behavior [24]. Several theoretical models suggest that cue-induced subjective craving and attentional bias reflect closely linked underlying processes [3,25,26], such that the degree of attentional bias toward reward cues correlates with the motivational, as opposed to the hedonic, qualities of the reward [27]. Clinically, attentional bias to smoking cues is linked to relapse following smoking cessation [28,29] and was found to be even more predictive of relapse than withdrawal symptoms, subjective craving, and low mood during acute abstinence [29]. Recently, the use of direct eye-tracking indices of attentional bias has shown substantial improvements in bias estimate reliability [30-33]. Assessment within naturalistic settings has also independently improved the reliability [34] and validity [35] of attentional bias measurement, yet the naturalistic constraints of these methods prohibit advanced clinical application. Thus, eye-tracking indices of attentional bias within naturalistic, yet clinically feasible settings, may be especially useful as biomarkers of the incentive salience/craving phenomenon in substance addiction.

Pupillary responses and EBR represent two lesser-studied eye characteristics with theoretical ties to incentive salience processes that warrant further study as potential biomarkers of addiction. Pupil diameter has been associated with engagement of cognitive resources [36], sensitivity to rewards [37], and reward processing broadly [38]. Pupil diameter changes indicate fluctuations in attention allocation and are suggested as a measure of attention-related constructs that do not reach the threshold of overt behavior or conscious appraisal [39]. Only one study has investigated pupillometry as a measure of response to substance cue exposure in humans and found that pupillary bias toward alcohol versus neutral cues, but not subjective craving reports, predicted relapse to alcohol use in a sample of detoxified patients with alcohol dependence [40].

EBR has been linked with striatal dopaminergic function in preclinical models and has been advanced by some as a reliable alternative to the assessment of dopaminergic functioning via positron emission tomography [41]. Dopamine release in the basal ganglia is theorized to inhibit the spinal trigeminal complex, consequently triggering increased EBRs [42]. Given the observed modulation of striatal dopamine during cue exposure [43], it may be possible to detect these dopaminergic fluctuations through EBR measurement. Yet, outside of our preliminary report on this sample [44], this hypothesis has not yet been tested.

This study sought to investigate the validity of these eye characteristics as markers of incentive salience acquired during a novel real-world VR nicotine and tobacco product (NTP) cue exposure paradigm across NTP users with varying degrees of use. An initial report was published by our group early on during data collection (N=31) [44] that described the development of the NTP cue VR paradigm and provided preliminary results supporting the potential of this paradigm as an effective lab-based cue exposure task, including its ability to elicit subjective craving and a sense of presence in the virtual world. The present study provides an update to this preliminary report with a larger sample of daily and nondaily users of NTPs (N=108). It was hypothesized that eye-based markers of attentional bias, pupillometry, and EBR would be greater in response to NTP cues compared with control cues presented during the VR NTP cue exposure paradigm and that these measures would correlate with subjective craving and measures of past NTP use.

## Methods

### Participant Recruitment and Screening Procedures

As previously described [44], participants were recruited through flyers and social media posts (eg, Facebook, Craigslist, and San Diego Reader) targeting the San Diego community. A brief telephone screening interview was used to determine initial eligibility. Inclusion criteria for the study were ages ≥18 years, at least weekly NTP use during the past 3 months, and NTP use history ≥1 year. Exclusionary criteria were nonfluency in English, medical or psychiatric history affecting brain development (ie, current severe *Diagnostic and Statistical Manual of Mental Disorders* [Fifth Edition; *DSM-5*] psychiatric disorders other than tobacco use disorders, severe head trauma with loss of consciousness >2 minutes, or history or treatment of neurologic disorders), and (3) visual problems that interfere with task completion (eg, severe motion sickness and blindness). NTP use was defined as use of any tobacco (eg, cigarette, cigar, or hookah) or electronic nicotine delivery system (eg, e-cigarette or vaporizer). NTP use groups were defined as daily users (average use of 7 days per week in the past 3 months) and nondaily users (average use of 4‐27 days per month in the past 3 months). The distinction between daily and nondaily users is supported by the literature, confirming that regular, voluntary, nondaily users of tobacco do not smoke often enough to regulate nicotine levels and evince less tobacco dependence and cue-induced craving as compared to daily users [45-47].

Eligible participants were invited into the laboratory and instructed to bring their NTPs with them for use immediately after the visit to control for effects related to expectations of imminent substance availability [48]. Participants were asked to abstain from NTP use for at least 1 hour prior to their visit, resulting in VR testing at least 2 hours post use (the average half-life of nicotine in body tissues [49]), and all other substance use (including alcohol and cannabis use) for at least 24 hours prior to testing. Abstinence was self-reported as COVID-19 restrictions did not allow for biological verification.

### Ethical Considerations

Participants received a detailed explanation of study procedures and provided written informed consent consistent with the University of California, San Diego Institutional Review Board policies upon arrival to the laboratory (UCSD IRB #180719). Participant data were deidentified. Participants received US $50 cash for completing the in-person session and up to US $60 in gift cards for completing the follow-up portion of the research (not presented here).

### Psychological and Substance Use Measures

Prior to undergoing the NTP cue VR paradigm, participants underwent a clinical interview to assess psychological health (Mini International Neuropsychiatric Interview for *DSM-5* [50]) and completed self-report questionnaires encompassing basic demographic information, previous VR experience, and other measures of psychological functioning not reported here.

The 90-day timeline follow-back (TLFB) [51] and Customary Drinking and Drug Use Record [52] interviews were administered to assess substance use history (including recency since last NTP use in minutes). The TLFB has high test-retest reliability for intervals ranging from 30 to 360 days prior to the interview date, with an intraclass correlation coefficient of 0.92 for “Total number of cigarettes smoked per interval” [53]. The Population Assessment of Tobacco and Health (PATH) tobacco dependence index [54], with a range of 0‐80, was administered to assess nicotine dependency across nicotine products. Subjective craving before and after the VR paradigm was assessed via the Tobacco Craving Questionnaire–Short Form (TCQ-SF) [55], modified to reference participants’ preferred nicotine product (eg, e-cigarettes and tobacco cigarettes). The TCQ-SF has demonstrated reliability (Cronbach α coefficients >0.69 across subscales) and validity, and has been shown to reliably measure the same multidimensional aspects of tobacco craving as the original TCQ when tested following overnight abstinence and during ad libitum smoking [55]. Pre- and post-VR TCQ-SF scores and previous 90-day NTP use episode count from the TLFB (logged transformed due to skewness) were used in the quantitative analyses presented below. REDCap electronic data capture tools hosted at the University of California, San Diego were used for interview and self-report data collection.

Following completion of the NTP cue VR paradigm, participants were assessed on VR presence (Igroup Presence Questionnaire [IPQ] [56]) and VR-related simulator motion sickness (Simulator Sickness Questionnaire [SSQ] [57]). The IPQ total score was calculated using a simple averaging method to obtain a perceived presence score ranging from 0 to 100. The SSQ was scored in concordance with procedures outlined to assess VR-specific sickness (Virtual Reality Sickness Questionnaire) [58], involving a simple averaging method to obtain a score ranging from 0 to 100.

### NTP Cue VR Paradigm

As previously detailed [44], the HTC VIVE Pro Eye VR headset (HTC, Taoyuan City, Taiwan) was used to enable VR capabilities and collect eye-related data during the NTP cue VR paradigm (built in Unity). HTC’s SRanipal SDK [59] was used in conjunction with Tobii’s XR SDK (Tobii Technology, Stockholm, Sweden) to provide access to data from the eye tracker. Specifically, Tobii’s XR SDK and Gaze-to-Object-Mapping (G2OM) algorithm were applied to determine object selections, while the remaining data were retrieved from the SRanipal SDK.

**Figure 1.**
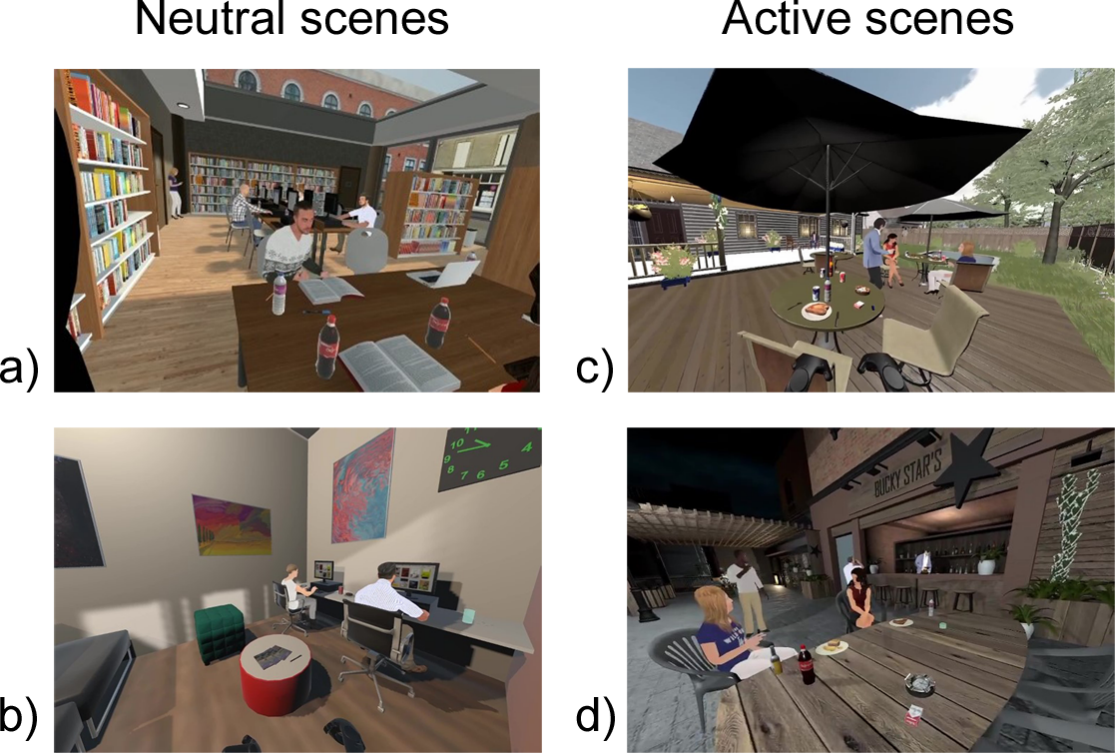
Screenshots of the four final scenes from the nicotine and tobacco product cue virtual reality paradigm. Neutral scenes include the (A) library and (B) waiting room. Active scenes include the (C) outdoor BBQ and (D) patio. The figure is adapted from the initial task development report [44], which is published under Creative Commons Attribution 4.0 International License [60].

Initially, 3 active scenes containing control and NTP-related cues (driving, patio, outdoor BBQ) and 3 neutral scenes containing only control cues (bus, waiting room, library) were developed. However, after preliminary testing of the paradigm, 1 active and 1 neutral scene were removed due to inconsistent eye-gaze effects and increased VR-related sickness (driving, bus; see Liu et al [44] for additional details). All active scenes contained multiple types of NTPs (see Liu et al [44] for a detailed description of the scenes). Thus, the data presented below are derived from the remaining 2 active (patio, outdoor BBQ) and 2 neutral (waiting room, library) scenes (Figure 1). Importantly, the selection of the study outcomes was done prior to any data acquisition and thus was not affected by removal of the scenes.

During the paradigm, participants were encouraged to move around in the virtual scenes via teleportation and interact with cue objects using two handheld VIVE controllers. Virtual visual analog scales assessing subjective craving (“How much are you craving nicotine right now?”) and scene relevance (“How relevant was that scene to your own life?”) were presented between scenes, and responses were made by adjusting a slide bar using one of the controllers. Participants were instructed to “Just explore everything around you until the scene changes.”

### Gaze Statistics Calculation

A combination of the G2OM algorithm provided by Tobii’s XR SDK, a machine learning–based mapping algorithm that aims to improve small object and fast-moving object tracking, and naive ray-casting was used to enable object selection in the direction of the gaze [61]. Specifically, to ensure adequate performance without detrimentally affecting the frame rate, the G2OM algorithm provided by Tobii’s XR SDK was used only for the detection of the interactable objects (including all NTPs and control cues), and the naive ray-casting was used for the detection of background and other nonmovable large objects. In addition, when a virtual object was interacted with via the controllers, the object selection was “locked” until the object was released, thus reducing eye-gaze errors due to rapid movement and microsaccades.

Given the complexity of the dynamic virtual environment, eye fixations were defined based on functionality—the duration of eye gaze intersection with the selected object of interest. The total object fixation number and total object fixation time (dwell time) were summed within each cue category (NTP and control) for each scene. Mean fixation time (total fixation time/object fixation number) indices were then created within each cue category for each scene and averaged across the scenes. Mean NTP versus control cue fixation and fixation time contrast scores from the active scene metrics were calculated for use in the exploratory analyses described below.

### Pupil Diameter and Blink Detection

Pupil diameter was recorded continuously throughout the paradigm and mapped to each object identified via Tobii’s G2OM algorithm. Pupil diameter was summed within each cue category (NTP and control) for each scene. Mean pupil diameter indices were then created by averaging over the mapped pupil diameter samples within each cue category for each scene and averaged across the scenes. Mean NTP versus control cue pupil diameter contrast scores (mean NTP cue diameter – mean control cue diameter) from the active scene metrics were calculated for use in exploratory analyses.

Consistent with previous studies, an eyeblink was defined as complete eyelid closure (or missing pupil diameter) with the pupil covered for 50‐500 milliseconds [62,63]. Total EBRs were summed within each scene and averaged within scene type (active and neutral). Mean active versus neutral scene EBR contrast scores (mean active scene EBR – mean neutral scene EBR) were calculated for use in exploratory analyses.

### Statistical Analysis

Statistical analyses for demographic differences between NTP use groups were conducted using one-way ANOVA models. Analyses for the main outcomes were conducted using repeated measures ANOVAs, followed by analyses of covariance controlling for age and sex. Interactions between NTP use group and cue/scene type as well as their main effects were estimated. Estimated marginal means (EMMs) are reported for the main effects that control for the other variable of interest (ie, NTP use group or cue/scene type) in the model. Analyses of preliminary reliability estimates across scenes were conducted using Pearson correlations. A significance threshold of *P*<.05 was set for all primary analyses.

Exploratory investigations of relationships between the objective outcomes (ie, total fixations, mean gaze fixation time, pupil diameter, and EBR) and subjective craving (pre-VR, during VR, post-VR), recency of NTP use (minutes since last NTP use at time of testing), and previous 90-day NTP use utilized Pearson correlations and partial correlations. Bonferroni-corrected *P* value thresholds that corrected for the tests of the 3 subjective craving and 2 NTP use variables per objective outcome were calculated (*P_corr_*<.01). Follow-up analyses computed correlations within NTP use groups, transformed the *r* values into *z* scores using Fisher *r*-to*-z* transformation, and compared the *z* values by determining the observed *z* test statistic. SPSS Statistics for Windows, version 28 (IBM Corp) software was used for all analyses.

## Results

### Study Sample

A total of 303 phone screenings were completed, with 193 individuals deemed eligible. The primary reasons for ineligibility were low/no NTP use (32 screenings) and severe psychiatric comorbidity/psychotropic medication use (39 screenings). Many eligible screenings were not enrolled due to COVID-19 restrictions/cancellations at the time. Of the 115 participants who completed the protocol, 108 participants had usable eye fixation data, 104 had pupillometry data, and 106 had EBR data (excluded participants had calibration or technical issues with the eye-tracking hardware/software).

Demographic information for the sample of 108 with eye fixation data is presented in Table 1. In general, the sample contained slightly more male participants (n=61, 56.5%) and predominately self-identified as White (n=60, 55.6%), and 58.3% (n=63) had no or very limited (one time) previous experience with VR. Of the full sample, 56.5% (n=61) were predominately e-cigarette or nicotine vaporizer users; however, 68.5% (n=74) of the sample reported smoking a tobacco cigarette, and 77.8% (n=84) reported use of any combustible tobacco product (cigarette, cigar, pipe, or hookah) within the previous 6 months. Daily and nondaily NTP use groups were not found to differ in type of NTP use (*P* values >.25).

**Table 1.** Sample demographics by nicotine and tobacco product (NTP) use group and total sample.

Variable		NTP use group		Total (N=108)
		Nondaily (n=33)	Daily (n=75)	
Age (years), mean (SD)		30.76 (12.58)	31.92 (12.75)	31.56 (12.65)
**Sex, n (%)**				
	Female	14 (42.4)	33 (44.0)	47 (43.5)
	Male	19 (57.6)	42 (56.0)	61 (56.5)
**Ethnicity: White, n (%)**		21 (63.6)	39 (52.0)	60 (55.6)
**Education: college level, n (%)**		31 (93.9)	63 (84.0)	94 (87.0)
**Previous VR^a^ experience, n (%)**				
	Never	17 (51.5)	26 (34.7)	43 (39.8)
	Once	5 (15.2)	15 (20.0)	20 (18.5)
	A few times	7 (21.2)	27 (36.0)	34 (31.5)
	Many times	4 (12.1)	7 (9.3)	11 (10.2)
Combustible tobacco product user (predominately), n (%)		18 (54.5)	29 (38.7)	47 (43.5)
NTP use days (previous 90 days)^b^, mean (SD)		33.91 (21.57)	89.79 (0.76)	72.71 (28.43)
NTP use episodes (previous 90 days)^b^, mean (SD)		161.82 (180.85)	2145.92 (2633.99)	1539.67 (2376.99)
PATH^c^ tobacco dependence index^b^, mean (SD)		15.94 (11.70)	46.15 (17.54)	36.92 (21.19)
Tobacco Craving Questionnaire (baseline)^b^, mean (SD)		30.09 (15.71)	47.55 (14.43)	42.17 (16.84)
**VR presence (IPQ^d^), mean (SD)**				
	Spatial presence	64.06 (18.16)	65.56 (17.80)	65.10 (17.84)
	Involvement	59.47 (21.30)	56.72 (22.16)	57.56 (21.84)
	Experienced realism	44.44 (23.07)	48.67 (22.39)	47.38 (22.58)
	Total	55.99 (8.65)	56.98 (9.60)	56.68 (9.29)
**VR-related sickness (VRSQ** ^**e**^ **), mean (SD)**				
	Oculomotor	18.43 (14.09)	17.22 (18.90)	17.59 (17.51)
	Disorientation	15.91 (14.64)	12.11 (17.67)	13.27 (16.82)
	Total	17.17 (13.21)	14.67 (17.25)	15.43 (16.10)

a VR: virtual reality.

b Denotes significant group differences (*P*<.001).

c PATH: Population Assessment of Tobacco and Health.

d IPQ: Igroup Presence Questionnaire.

e VRSQ: Virtual Reality Sickness Questionnaire.

### Subjective Craving

**Figure 2.**
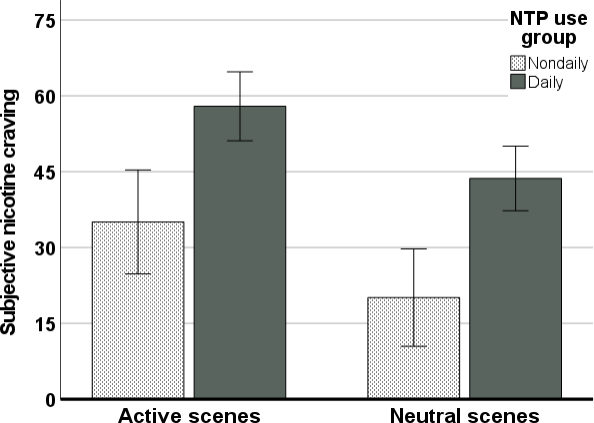
Mean subjective craving ratings averaged across active and neutral scenes by NTP use group. Error bars indicate a 95% CI. NTP: nicotine and tobacco product.

Results of the ANOVA investigating subjective craving during the paradigm revealed significant effects of scene condition (*F*_1,106_=47.95; *P*<.001; η_p_^2^=0.31) and NTP use group (*F*_1,106_=16.91; *P*<.001; η_p_^2^=0.14) on craving. No interaction between scene condition and NTP use group was observed (*F*_1,106_=0.03; *P*=.87; η_p_^2^<0.001). Specifically, active scenes (EMM 46.50, SE 3.11) elicited greater subjective craving than neutral scenes (EMM 31.89, SE 2.92; Figure 2), and daily users reported greater subjective craving across scenes (EMM 50.81, SE 3.12) than nondaily users (EMM 27.59, SE 4.70). Controlling for age and sex in this analysis reduced the main effect of scene condition (*F*_1,104_=4.16; *P*=.04; η_p_^2^=0.04) but not NTP use group (*F*_1,104_=17.63; *P*<.001; η_p_^2^=0.15). Age and sex were not found to be significant predictors of subjective craving either via direct effects or interactions (*P* values >.05). Subjective craving ratings were found to positively correlate between the ratings following the 2 active scenes (*r*=0.844; *P*<.001) and between the ratings following the 2 neutral scenes (*r*=0.816; *P*<.001).

### Total Cue Eye-Gaze Fixations

Results of the ANOVA investigating total cue eye-gaze fixations during the paradigm revealed a significant effect of cue type on number of fixations during the active scenes (*F*_1,106_=1353.18; *P*<.001; η_p_^2^=0.93). No effect of NTP use group (*F*_1,106_=0.03; *P*=.85; η_p_^2^<0.001) or interaction between cue type and NTP use group (*F*_1,106_<0.001; *P*=.98; η_p_^2^<0.001) were observed. Specifically, NTP cues were associated with fewer total fixations (EMM 12.91, SE 0.21) as compared to control cues (EMM 48.11, SD 1.00). Controlling for age and sex in this analysis reduced, but did not eliminate, the main effect of cue type (*F*_1,104_=175.04; *P*<.001; η_p_^2^=0.63). Further, there was a significant interaction observed between cue type and sex (*F*_1,104_=8.40; *P*=.005; η_p_^2^=0.07), whereby male participants engaged in more total fixations toward control cues (EMM 50.07, SE 1.19) as compared to female participants (EMM 45.67, SE 1.35), yet their total fixations toward NTP cues were similar (male participants: EMM 12.69, SE 0.24; female participants: EMM 13.28, SE 0.28). Borderline main effects of age (*F*_1,104_=3.69; *P*=.06; η_p_^2^=0.03) and sex (*F*_1,104_=3.81; *P*=.05; η_p_^2^=0.03) were also observed. When comparing the 2 active scenes, total cue eye-gaze fixations were found to positively correlate for the NTP cues (*r*=0.347; *P*<.001) and control cues (*r*=0.657; *P*<.001).

### Mean Eye-Gaze Fixation Time (Attentional Bias)

**Figure 3.**
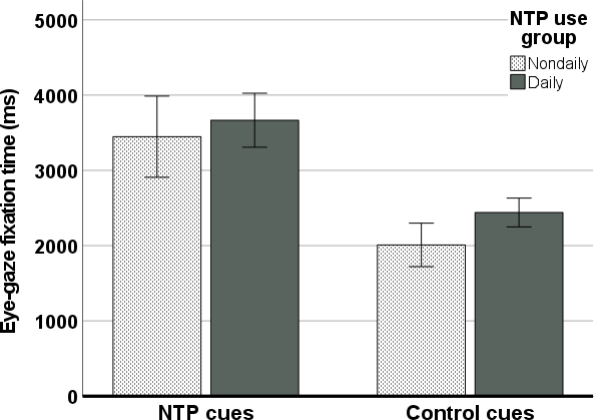
Mean eye-gaze fixation time averaged across NTP and control cues by NTP use group. Error bars indicate a 95% CI. NTP: nicotine and tobacco product.

Results of the ANOVA investigating mean eye-gaze fixation time (attentional bias) during the paradigm revealed a significant effect of cue type on fixation time during the active scenes (*F*_1,106_=48.34; *P*<.001; η_p_^2^=0.31) and some support for a NTP use group effect (*F*_1,106_=3.31; *P*=.07; η_p_^2^=0.03; Figure 3). No interaction between cue type and NTP use group was observed (*F*_1,106_=0.31; *P*=.58; η_p_^2^=0.003). Specifically, NTP cues were associated with greater mean fixation times (EMM 3557.79, SE 163.36 ms) as compared to control cues (EMM 2225.54, SE 87.34 ms), and daily users demonstrated greater mean fixation times across cue type (EMM 3054.09, SE 98.75) as compared to nondaily users (EMM 2729.24, SE 148.87). Controlling for age and sex in this analysis reduced, but did not eliminate, the main effect of cue type (*F*_1,104_=14.32; *P*<.001; η_p_^2^=0.12), and the NTP use group effect was largely unchanged (*F*_1,104_=3.53; *P*=.06; η_p_^2^=0.03). Age and sex were not found to be significant predictors of mean eye-gaze fixation time either via direct effects or interactions (*P* values >.05). When comparing the 2 active scenes, mean eye-gaze fixation times were found to positively correlate for the NTP cues (*r*=0.261; *P*=.006) and control cues (*r*=0.462; *P*<.001).

### Pupil Diameter

**Figure 4.**
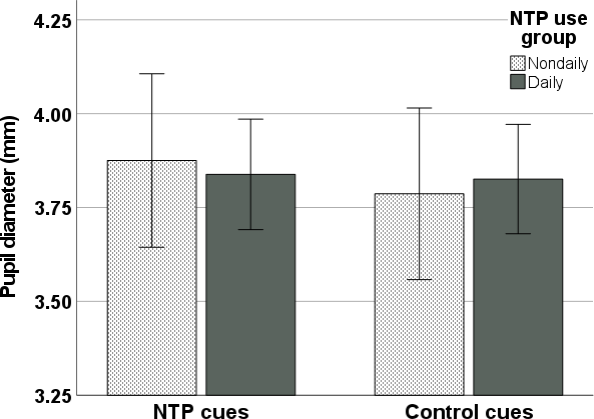
Mean pupil diameter averaged across NTP and control cues by NTP use group. Error bars indicate a 95% CI. NTP: nicotine and tobacco product.

Results of the ANOVA investigating mean pupil diameter during the paradigm revealed a significant effect of cue type on mean pupil diameter during the active scenes (*F*_1,102_=5.99; *P*=.02; η_p_^2^=0.05) and some support for an interaction between cue type and NTP use group (*F*_1,102_=3.38; *P*=.07; η_p_^2^=0.03; Figure 4). No NTP use group main effect was observed (*F*_1,102_<0.001; *P*=.99; η_p_^2^<0.001). Specifically, NTP cues were associated with greater pupil diameter (EMM 3.86, SE 0.07 mm) as compared to control cues (EMM 3.81, SE 0.07 mm) across groups, but only the nondaily use group displayed a significant difference between cue types (nondaily mean difference 0.09, SE 0.03; *P*=.01; daily mean difference 0.01, SE 0.02; *P*=.57). Controlling for age and sex in this analysis reduced the main effect of cue type (*F*_1,100_=2.25; *P*=.14; η_p_^2^=0.02) and the interaction between cue type and NTP use group (*F*_1,100_=3.26; *P*=.07; η_p_^2^=0.03). A main effect of age on pupil diameter was also observed (*F*_1,100_=18.36; *P*<.001; η_p_^2^=0.16). When comparing the 2 active scenes, pupil diameters were found to positively correlate for the NTP cues (*r*=0.793; *P*<.001) and control cues (*r*=0.765; *P*<.001).

### Spontaneous Eyeblink Rate

Results of the ANOVA investigating mean EBR found no significant differences between EBR during active and neutral scenes (*F*_1,104_=0.50; *P*=.48; η_p_^2^=0.005) or between NTP use groups (*F*_1,104_=0.17; *P*=.68; η_p_^2^=0.002), nor a significant interaction (*F*_1,104_=0.37; *P*=.54; η_p_^2^=0.004). Controlling for age and sex in this analysis resulted in no change to the relationships. Age and sex were not found to be significant predictors of EBR either via direct effects or interactions (*P* values >.05). EBRs were found to positively correlate between the 2 active scenes (*r*=0.635; *P*<.001) and between the 2 neutral scenes (*r*=0.567; *P*<.001).

### Relationship to NTP Subjective Craving and Use

**Figure 5.**
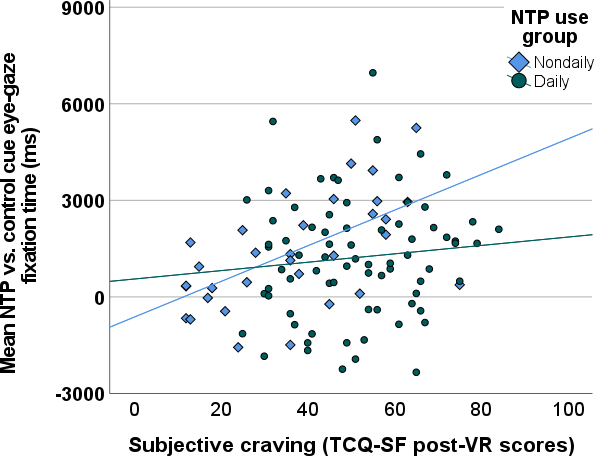
Scatterplot depicting the linear relationships between mean NTP versus control cue eye gaze fixation time contrast scores (in milliseconds) from the active scenes and subjective craving from the TCQ-SF administered post-VR paradigm by NTP use group. NTP: nicotine and tobacco product; TCQ-SF: Tobacco Craving Questionnaire—Short Form; VR: virtual reality.

Mean NTP versus control cue fixation time contrast scores (attentional bias scores) were found to positively correlate with subjective craving assessed within the paradigm (*r*=0.19; *P*=.04) and with the TCQ-SF administered pre- (*r*=0.18; *P*=.06) and post-VR paradigm (*r*=0.22; *P*=.02). Comparison of correlations between NTP use groups demonstrated a significant group difference (*Z*_obs_=2.48; *P*=.007), with the nondaily group demonstrating a stronger positive correlation (*r*=0.57; *P*=.001) compared to the daily group (*r*=0.10; *P*=.38) in TCQ-SF scores post-VR paradigm (Figure 5). Similar group relationships held for the other craving metrics. Follow-up analyses investigating mean cue fixation time separately by cue type (NTP and control) in the full sample revealed that the positive correlations with all three subjective craving ratings were driven primarily by mean NTP cue fixation times (*r* values=0.21-0.28; *P* values <.03), as opposed to control cue fixation times (*r* values=0.02-0.03; *P* values >.76). The mean NTP versus control cue fixation time contrast score (attentional bias) was not found to correlate with previous 90-day NTP use (*r*=0.05; *P*=.58), yet significant positive correlations with previous 90-day NTP use were observed for mean NTP cue fixation time (*r*=0.20; *P*=.03) and control cue fixation time (*r*=0.26; *P*=.007) when analyzed independently.

Mean NTP versus control cue pupil diameter contrast scores were not found to correlate with subjective craving measures across the full sample (*P* values >.20) or NTP use groups independently (*P* values >.07). Mean NTP versus control cue pupil diameter was found to negatively correlate with previous 90-day NTP use (*r*=–0.20; *P*=.04), yet no significant correlations with previous 90-day NTP use were observed for the NTP (*r*=0.09; *P*=.37) or control cue (*r*=0.15; *P*=.12) pupil diameters independently.

Partial correlations controlling for age and sex resulted in similar estimates for all analyses described above except for the correlation between mean NTP versus control cue pupil diameter contrast scores and previous 90-day NTP use, which was eliminated when age and sex were controlled for (*r_partial_*=−0.05; *P*=.64). Total cue fixations and eyeblink rates were not found to correlate with any subjective craving measures or previous 90-day NTP use (*P* values >.05). None of the objective measures significantly correlated with recency of NTP use (*P* values >.05). None of the first-level correlations survived multiple comparison correction (Bonferroni-corrected per objective measure *P* values <.01) and, as such, must be interpreted with caution.

## Discussion

This study provides updated results on the utility of a novel VR NTP cue exposure paradigm to index incentive salience via three eye characteristic markers: eye-gaze fixation time (attentional bias), pupil diameter, and EBR in response to NTP versus control cues. Overall, the results are largely consistent with our preliminary report [44] and support two of our initial hypotheses, suggesting that measures of eye-gaze fixation time and pupil diameter, but not EBR, during VR cue exposure could be useful objective indicators of the incentive salience process in nicotine addiction.

Consistent with previous VR cue exposure investigations across a variety of substances [14-21], active VR scenes with NTP cues elicited greater subjective craving compared to neutral control scenes. Further, daily NTP users endorsed greater overall levels of subjective craving compared to nondaily users across scenes. Together these findings suggest the VR NTP cue exposure paradigm elicits subjective phasic craving in response to NTP cues and can discriminate by frequency of NTP use on this metric.

Given that the intention of the paradigm was to provide a more naturalistic and translatable context of use than standard cue exposure and attentional bias paradigms, it follows that more control versus NTP cues were present in the active scenes. As a result, greater total eye-gaze fixations toward control versus NTP cues were observed. Yet, the average gaze fixation time was found to be 1.33 seconds longer for the NTP cues compared with the control cues across the full sample, thus demonstrating attentional bias toward the NTP cues regardless of NTP use frequency. The attentional bias contrast score (mean NTP vs control cue fixation time) was also modestly, yet consistently, associated with measures of subjective craving assessed before, during, and after the VR paradigm. This was primarily driven by response to the NTP cues, as opposed to the control cues, supporting the previously established link between attentional bias, as indexed by fixation time, and subjective craving [10,30,64-66]. The culmination of fixation time results also supports the validity of the VR NTP cue exposure paradigm as suitable for measuring attentional bias toward NTP cues in a free-viewing, translatable, and ecologically valid context.

Interestingly, although no interaction between NTP use group and cue type was observed, the daily NTP users were found to fixate on all cues (NTP and control) longer (325 ms) than the nondaily users. Furthermore, no association was observed between the attentional bias contrast score and previous NTP use frequency, yet greater mean gaze fixation time to NTP and control cues were *independently* associated with greater NTP use in the previous 90 days. These results are somewhat contradictory to the findings by Mogg and colleagues [67], where greater smoking versus control fixation times were inversely associated with nicotine dependence. This discrepancy could relate to differences in tasks, as Mogg et al [67] used a visual probe task to assess eye-gaze fixation time, which presented cues in isolation, devoid of context and additional competing cues. Additionally, independent associations between cue types and dependence severity were not reported in their paper; thus, it remains unknown whether a similar relationship would have been observed in their data. Regardless, our results suggest that in the presence of additional naturalistic context and the absence of any researcher-directed task demands, individuals with varying levels of nicotine dependence evince attentional bias toward NTP cues and more frequent/dependent NTP users demonstrate prolonged attentional engagement with all salient visual cues present.

Consistent with Mogg and colleagues [67] and with subjective craving associations broadly [68], we observed stronger correlations between the attentional bias contrast score and subjective craving levels within the nondaily NTP users, as compared to the daily users. Relatedly, greater pupillary diameter was observed in response to NTP cues compared to control cues, particularly within the nondaily users. Interestingly, the NTP versus control cue pupillary response contrast was found to be negatively associated with previous NTP use, although these effects were essentially eliminated after controlling for age, a known correlate of pupil size [69]. These findings are in line with theories suggesting that appetitive motivational processes (ie, incentive salience) reduce in importance as addiction becomes more severe and habitual [5,6,67].

Taken together with our attentional bias and NTP use data, there may be additional nonselective attentional processes occurring in individuals with more severe nicotine dependence that are not routinely captured by traditional subjective and procedural tasks of attention. For example, our results may reflect an effect of prolonged nicotine use on general attentional processing in the absence of task demands and trial durations, whereby individuals with more prolonged NTP use may have delayed disengagement from any salient cue in their visual environment. These correlations appear to hold even after controlling for total number of cue fixations, suggesting this is not a product of orientation bias. Traditional tasks used to investigate attentional bias (eg, Stroop and visual probe tasks) are thought to index the delayed disengagement of attention; yet their ability to do so is limited by trial carryover effects, short durations of stimulus onset asynchrony (SOA), and task demands (eg, to shift attention based on cue location [24]). Even with tasks thought to explicitly measure disengagement, the SOA is often only 500‐2000 milliseconds [24], yet our data suggest that when free-viewing a complex scene with many cues present, individuals spend on average 2955 milliseconds engaged and attending to one object cue irrespective of NTP use history and cue type. Thus, further investigations using these paradigms to assess attentional disengagement may benefit from increasing SOA beyond 3000 milliseconds to ensure they are capturing the entire disengagement process. Given that acute nicotine administration facilitates attention disengagement from a cued location [70], it may be possible that the reverse effect is occurring during the state of acute withdrawal in heavier users of NTPs.

This study has several strengths and limitations. Strengths include the inline assessment of eye characteristics during a translatable real-world VR NTP cue exposure paradigm with no imposed task demands, thus more accurately indexing naturalistic attentional and incentive salience processes. The inclusion of light to heavy users of various NTPs and ages increases the generalizability of the findings to the majority of current nicotine users. Limitations include the absence of biological verification to confirm self-reported NTP use due to COVID-19–related precautions, absence of prospective NTP use data, and the short duration of abstinence at the time of testing. However, substantial variability in abstinence was reported and abstinence time was not found to substantially impact the results. Still, studies investigating these effects at much longer durations of abstinence and in treatment-seeking populations may observe differing results as the salience of cues may change based on extended abstinence. Given that the active scenes differ in the amount and nature of the cues (both NTP and control), additional studies with multiple identical administrations and with prospective NTP use data are needed to adequately assess the reliability and validity of these eye-tracking indices. Lastly, given the relationship between increasing age and potential for greater addiction severity (eg, allowing for greater years of use with increasing age), future studies are needed to identify the independent contributions of age and eye-related variables (especially pupil size [69]) on NTP use outcomes.

In summary, this study represents an update to our initial paper [44], provides validation of the utility of the VR NTP cue exposure paradigm for the assessment of attentional bias as measured via eye-gaze fixation time and pupillometry, and highlights areas for further consideration in other attentional bias paradigms (eg, increasing SOA). Given that attentional bias has been shown to predict relapse following smoking cessation [28], these markers may prove useful in clinical settings by facilitating the matching of individuals who exhibit greater attentional bias with interventions targeting incentive salience processes (eg, varenicline [16,18] and mindfulness [71]). The validation of reliable biomarkers of addiction such as attentional bias could also greatly benefit treatment development by providing an earlier identification of treatment efficacy (a “fast fail” marker) in clinical trials. Broadly, markers such as attentional bias and pupil diameter have the potential to provide much needed objective measures of addiction phenotypes, thus reducing error associated with phenotyping and outcomes measurement based solely on subjective assessments.

## Abbreviations

*DSM-5*: *Diagnostic and Statistical Manual of Mental Disorders* (Fifth Edition)
EBR: spontaneous eyeblink rate
EMM: estimated marginal mean
G2OM: Gaze-to-Object-Mapping
IPQ: Igroup Presence Questionnaire
NTP: nicotine and tobacco product
PATH: Population Assessment of Tobacco and Health
SOA: stimulus onset asynchrony
SSQ: Simulator Sickness Questionnaire
TCQ-SF: Tobacco Craving Questionnaire–Short Form
TLFB: timeline follow-back
VR: virtual reality
